# Potential diagnostic markers and therapeutic targets for rheumatoid arthritis with comorbid depression based on bioinformatics analysis

**DOI:** 10.3389/fimmu.2023.1007624

**Published:** 2023-02-23

**Authors:** Tao-tao Zhou, Ji-jia Sun, Li-dong Tang, Ying Yuan, Jian-ying Wang, Lei Zhang

**Affiliations:** ^1^ Department of Mathematics and Physics, School of Pharmacy, Shanghai University of Traditional Chinese Medicine, Shanghai, China; ^2^ Teaching and Research Section of Chinese Materia Medica, School of Pharmacy, Shanghai University of Traditional Chinese Medicine, Shanghai, China; ^3^ Shanghai Innovation Center of Traditional Chinese Medicine Health Service, Shanghai University of Traditional Chinese Medicine, Shanghai, China

**Keywords:** rheumatoid arthritis, depression, bioinformatics, machine learning, molecular docking

## Abstract

**Background:**

Rheumatoid arthritis (RA) and depression are prevalent diseases that have a negative impact on the quality of life and place a significant economic burden on society. There is increasing evidence that the two diseases are closely related, which could make the disease outcomes worse. In this study, we aimed to identify diagnostic markers and analyzed the therapeutic potential of key genes.

**Methods:**

We assessed the differentially expressed genes (DEGs) specific for RA and Major depressive disorder (MDD) and used weighted gene co-expression network analysis (WGCNA) to identify co-expressed gene modules by obtaining the Gene expression profile data from Gene Expression Omnibus (GEO) database. By using the STRING database, a protein–protein interaction (PPI) network constructed and identified key genes. We also employed two types of machine learning techniques to derive diagnostic markers, which were assessed for their association with immune cells and potential therapeutic effects. Molecular docking and *in vitro* experiments were used to validate these analytical results.

**Results:**

In total, 48 DEGs were identified in RA with comorbid MDD. The PPI network was combined with WGCNA to identify 26 key genes of RA with comorbid MDD. Machine learning-based methods indicated that RA combined with MDD is likely related to six diagnostic markers: *AURKA*, *BTN3A2*, *CXCL10*, *ERAP2*, *MARCO*, and *PLA2G7*. *CXCL10* and *MARCO* are closely associated with diverse immune cells in RA. However, apart from *PLA2G7*, the expression levels of the other five genes were associated with the composition of the majority of immune cells in MDD. Molecular docking and *in vitro* studies have revealed that Aucubin (AU) exerts the therapeutic effect through the downregulation of *CXCL10* and *BTN3A2* gene expression in PC12 cells.

**Conclusion:**

Our study indicates that six diagnostic markers were the basis of the comorbidity mechanism of RA and MDD and may also be potential therapeutic targets. Further mechanistic studies of the pathogenesis and treatment of RA and MDD may be able to identify new targets using these shared pathways.

## Introduction

Rheumatoid arthritis (RA) is a prevalent chronic autoimmune disease, which affects around 0.5–1% of the world’s population (1). RA is primarily characterized by joint inflammation and symmetrically active polyarthritis. These conditions affect the metacarpophalangeal joints and result in stiffness, pain, and swelling of the joints (2). It is well known that inflammatory cascades of patients with RA can be initiated or exacerbated by genetic and certain environmental factors (3). Inflammation in patients with RA can lead to systemic responses, including endothelial dysfunction (4). Therefore, RA shares a tight relationship with a number of illnesses, such as diabetes, depression, and myocardial infarction (5, 6). Depression is a common mental illness, affecting 1.5–19.0 in 1,000 adults (7). According to epidemiological research, the proportion of RA patients who also experience comorbid depressive symptoms is 13–20%, which is around three times greater than that of the general populace (8). Another study revealed that the likelihood of developing RA was 1.7 times higher in patients with depression than in controls (9). There is strong evidence that RA and depression are related by mutually influencing each other; RA can lead to MDD and MDD can exacerbate RA. The emergence of a large number of controlled clinical trials of psychotherapy for RA has demonstrated that treating depression is an effective way to improve RA independent of drug treatment (10). Therefore, screening and treatment of depression in patients with RA has important clinical significance.

Similar to many other chronic pain diseases, pain and physical impairment in people with RA as the chronic disease progresses are often cited as the cause of Major depressive disorder (MDD) (11). However, patients with RA experience substantially more symptoms of depression than patients with osteoarthritis, even though pain and dysfunction are similar between the two diseases. This difference may be related to cytokine-related neuroimmunobiological mechanisms (12). Many cytokines, including IL-1, TNF-α, and IL-6, are secreted during the pathological process of RA. These cytokines have been linked to neuroinflammation in the brains of individuals with depression (13–15). Abnormal activation of monoamine neurotransmitters is now recognized as playing a decisive role in the pathogenesis of depression. Cytokines access the brain directly or indirectly, disrupt the metabolism of monoamine neurotransmitters, alter the body’s mental and cognitive activities, and lead to depression (16). Proinflammatory cytokines activate serotonin- and tryptophan-degrading enzymes while increasing the creation of glutamate-N-methyl-D-aspartate receptor agonists in the humoral immune system, resulting in serotonin deficiency and glutamate acid overproduction, both of which contribute to depression (17). Furthermore, by reducing the levels of neurotrophic factors in the brain, inflammatory factors may influence neurogenesis and neuroplasticity (18).

RA is highly inheritable, with approximately 60% heritability observed in twin studies (19). Approximately 100 loci that are significantly associated with RA have been identified in the genome. Additionally, a number of RA susceptibility genes have been linked to disease severity (20). Many alleles are weakly associated with RA, but the cumulative effects are observed in the presence of multiple risk alleles (21). It is important to investigate the multi-omics correlation in RA patients with depression, however, there have been no genomic studies on RA associated with depression. It is worth noting that Azathioprine, a Rac1 inhibitor, which is an immunosuppressant commonly used as adjunctive therapy for RA, have been reported to increase the risk of depression (22). Recently, there have been increasing reports of the use of natural products for the treatment of RA combined with depression. *Morinda officinalis* is often used in China because of its anti-osteoporosis, antidepressant, anti-Alzheimer disease, anti-rheumatoid, anti-oxidation, anti-inflammation, and anti-fatigue effects. The crude extracts and pure compounds of this plant are mainly composed of polysaccharides, anthraquinones, iridoid glycosides, and oligosaccharides. More than 100 chemical compounds have been isolated from *M. officinalis* that have been shown to have promising therapeutic effect on depression, osteoporosis, fatigue, and RA (23). Xinfeng Capsule (XFC) is an new effective natural medicine for the treatment of RA (24). In clinical studies, disease activity indexes, number of joint swelling/tenderness, joint morning stiffness duration, and all apoptosis-related indicators were reduced in the XFC group and the leflunomide group after treatment. However, XFC, which is composed of natural products, showed greater improvement on the self-rated depression scale than the leflunomide group (25).

In this study, we explored the common genes between RA and depression to reveal the underlying biological processes in RA combined with depression. This study aimed to explore the common genes of RA and depression to reveal the underlying biological processes in RA combined with depression. Diagnostic markers were identified from common genes to study their association with immune infiltration and their potential as diagnostic biomarkers and therapeutic targets.

## Materials and methods

### Data processing and analysis

**Figure 1:**
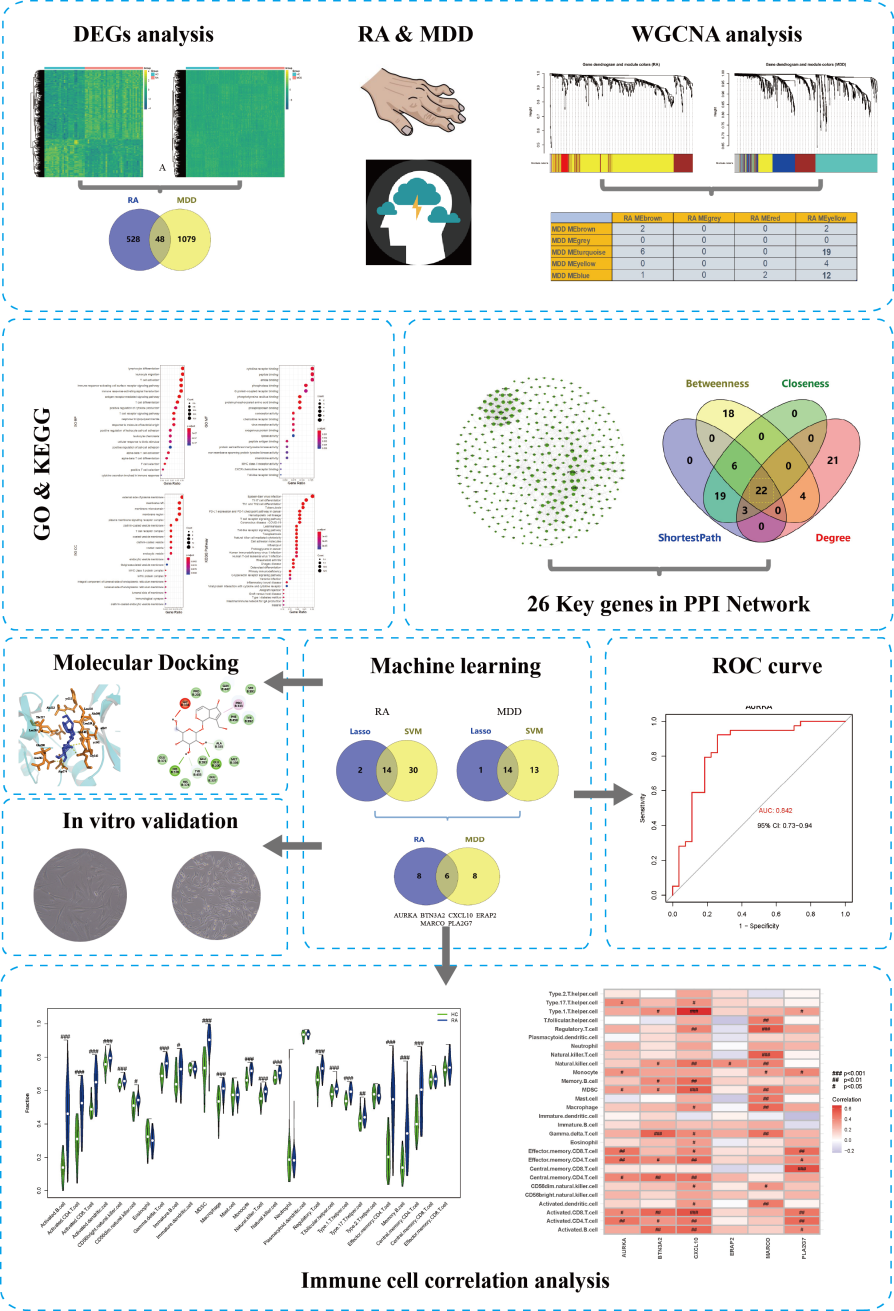
The complete research workflow.

We downloaded the GSE55235 (26), GSE55457 (26), and GSE77298 (25) RA datasets from the Gene Expression Omnibusdatabase (https://www.ncbi.nlm.nih.gov/geo/) using RStudio software (version 4.0.2; URL: https://www.r-project.org/). All dataset processing and analysis were performed in RStudio. The GSE55235 dataset (GPL96 platform), which was uploaded in 2014, contains transcriptome analyses of synovial tissue from 10 RA patients and 8 individuals with healthy joints. The GSE55457 dataset (GPL96 platform) identified 13 synovial membrane samples from patients with RA and 10 normal control synovial membrane samples. The samples in GSE55235, GSE55457 datasets were obtained from patients with RA for more than ten years. The RA dataset GSE77298 (GPL570 platform) contained a total of 23 synovial samples, which were obtained from 16 RA patients and 7 healthy individuals. In this dataset, the synovial samples were obtained from early RA at the Department of Rheumatic. Depression-associated transcriptomes (GSE98793) were obtained from the GEO database. In the GSE98793 dataset (27) (GPL570 platform), whole blood from 128 patients with MDD samples and 64 healthy individuals was collected. MDD was defined as CORE score >=8. According to the different sources of the samples, we categorized the samples into the RA group, depression group, and normal group, respectively. A simplified workflow of the current investigation is presented in **Figure 1**.

### Differentially expressed gene analysis

We used the limma package (version 3.44.0) of R (version 4.0.2) to standardize and correct all gene expression profiling microarray data and annotated the gene names. The SVA package (version 3.36.0) was used to remove batch effects. The RA gene expression profiling dataset, which contained 27 normal control samples and 39 RA samples, was used to analyze the differentially expressed genes (DEGs). The MDD gene expression profiling dataset was preprocessed in the same manner. A conservative threshold (|log2FC| > 1.0, *p* < 0.05) was used to screen for DEGs in patients with RA or MDD. The intersection genes between the DEGs of RA and MDD were generated using an online Venn diagram generator (version 2.1.0; https://bioinfogp.cnb.csic.es/tools/venny).

### Construction of co-expressed gene modules

Based on the DEGs of RA and MDD, which were screened using the threshold, we further applied weighted gene co-expression network analysis (WGCNA) to define functional transcriptomic co-expression modules shared by RA and MDD. To identify co-expression modules, the WGCNA package (28) (version 1.71) in R was used to create unsigned co-expression networks. To begin with, the flashClust program in R was used to perform a hierarchical clustering analysis of the samples to discover and eliminate outliers. Second, a “soft” thresholding power (β), generated by the WGCNA’s “pickSoftThreshold” algorithm, was utilized to design a physiologically important scale-free network according to the scale-free topology criterion. Third, a dynamic tree-cutting technique was used to create a topological overlap matrix (TOM) based on the adjacency matrix to detect gene modules. Fourth, gene significance (GS) and module membership (MM) were determined for linking modules to clinical features. Finally, we constructed the eigengene network.

### Protein–protein interaction network analysis

The STRING database (29) (version 11.0; https://string-db.org/) was used to construct a protein–protein interaction (PPI) network of co-expressed gene modules in RA with comorbid MDD. The parameter settings for the network construction were as follows: organism, Homo sapiens; combined score threshold, 0.7. The PPI network was visualized using the Gephi software (version 0.9.2; https://gephi.org/). Key genes (highly connected genes) were selected using the Cytoscape (30) (version 3.7.1; https://cytoscape.org/) plugin network analyzer (31). The network level (average shortest path length and betweenness centrality) and node level (network degree value and closeness centrality) topological features of the network were calculated. The four network properties reflect the importance of each protein node in the network, we screened out the shared proteins of RA and MDD, which were the top 50 proteins in the PPI network’s four network properties. Shared protein-coding genes are the key genes for RA associated with MDD.

### Functional enrichment analysis of core genes

The core gene set of RA associated with MDD was composed of DEGs and key genes obtained from PPI network analysis. The primary goals of this research were to identify the comorbidity mechanisms that link RA and MDD as well as to reveal the underlying molecular biological processes of the disease core genes. Gene Ontology (GO) and Kyoto Encyclopedia of Genes and Genomes (KEGG) pathway enrichment analyses were used to identify the characteristic biological and functional attributes (32, 33). GO and KEGG analyses were performed using the clusterProfiler package (34) (version 3.14.3) in R. A *p*-value of < 0.05 and a *q*-value of < 0.05 were reserved, and a higher Gene Ratio was considered more significant.

### Machine learning methods for the discovery of diagnostic markers

We used a machine learning approach to predict disease-associated genes based on the core genes of RA with comorbid MDD. In this study, two types of machine learning approaches were applied in the process of feature selection and model training: the LASSO regression model and support vector machine (SVM) method. The SVM algorithm was implemented using the caret package (version 6.0-86), kernlab package (version 0.9-29), and e1071 package (version 1.7-9). Ten-fold cross-validation was applied to calculate the misclassification error of our model within the training cohort. To calculate the misclassification error in the training cohort, a ten-fold cross-validation was applied to obtain the accuracy of the model algorithm. We first obtained the diagnostic markers in the RA and MDD datasets, and the overlapping part of the diagnostic markers of the two diseases represented the diagnostic markers in patients with RA and MDD.

### Diagnostic core genes and immune cell correlation analysis

The single sample Gene Set Enrichment Analysis (ssGSEA) was applied to explore the relationship between different infiltration degrees of immune cell types and the diagnostic markers of RA with comorbid MDD using the R package “GSVA” (version 1.44.0). By comparing the differences between the groups and the correlation between diagnostic marker expression and immune cell content, we aimed to investigate the link between diagnostic markers and immune cells.

### Molecular docking analysis

*Eucommia ulmoides Oliver* (EUO) has a long history of medicinal use in China. As a medicinal plant used for tonifying kidney, strengthening bones, relieving pain, and enhancing immunity, EUO is also widely used in the treatment of RA, depression, and osteoporosis. The aqueous extract of EUO has been demonstrated to have a cartilage-protecting effect in a rat model of osteoarthritis, potentially by inhibiting chondrocyte apoptosis and improving cartilage metabolism (35). Aucubin (AU), an iridoid glycoside that is an active constituent of EUO, has been extensively studied for the management of neurological diseases (36). However, a comprehensive review of its effects and mechanisms is currently unavailable. Therefore, in this study, we investigated the therapeutic potential of AU. The utilization of molecular docking, a technique commonly employed in virtual screening studies, was carried out to identify potential therapeutic targets for AU (37).

Primarily, the cheminformatics of Aucubin (AU) was obtained from the PubChem database (38) (https://pubchem.ncbi.nlm.nih.gov/), which included chemical name, molecular formula, CAS, PubChem CID, canonical SMILES, and SDF files. The ACD/Labs software (https://www.acdlabs.com/), SwissADME online system (39) (http://www.swissadme.ch/) and ADMETlab 2.0 (https://admetmesh.scbdd.com/) (40)were used to evaluate the pharmacokinetics and safety profile of AU, including absorption, distribution, metabolism, excretion, and toxicity. PyMOL software (version 1.7.0; https://pymol.org/) converted AU’s 3D structure, which was downloaded from the PubChem database (41) (http://www.rcsb.org/), from an SDF file to a PDB file while minimizing the energy of small molecules and then saved it as a PDBQT format file. The 3D structures of potential targets were downloaded from the PDB database (http://www.rcsb.org/). PyMOL software removed water molecules and hetero-ions from the PDB file of the target protein. The protein ligands then underwent hydrogenation and the charge was added in AutoDockTools (42) (version 1.5.6) software. Finally, the data were saved as a PDBQT file. The docking box parameters were determined based on the binding region of the protein receptor and original ligand, and the box size was set to 30Å × 30 Å × 30 Å. AutoDock Vina (version 1.1.2; http://vina.scripps.edu/) software performs refined the semi-flexible molecular docking and calculated the affinity (kcal/mol) of all potential key targets for AU. Generally, the lower the affinity value, the stronger the binding of the small molecule to the receptor. Discovery Studio Visualizer (https://www.3ds.com/) was used to visualize the 2D schemes of the AU-target protein interaction.

### Cell culture and MTT assay

Rat adrenal pheochromocytoma cells (PC12) and human rheumatoid fibroblast-like synoviocytes (HFLS) were obtained from iCell Bioscience Inc. and JENNIO Biological Technology, respectively. PC12 cells were cultured in 1640 basal medium containing 10% fetal bovine serum (FBS) and 1% Penicillin-Streptomycin, while HFLS cells were cultured in DMEM basal medium with the same supplements. The cells were incubated at 37°C in an atmosphere containing 5% CO2. PC12 cells were seeded at a density of 2x10^4^ cells/well and incubated for 24 hours before exposure to AU. Different concentrations of AU (ranging from 0 to 160 μM) were then added to the wells and incubated for an additional 24 hours. MTT solution (10 μM) was added to each well and incubated for a further 4 hours. The medium was then removed and DMSO (200 μl) was added to dissolve the formazan crystals formed by the viable cells. The absorbance at 490 nm was measured using a microplate reader. To evaluate the effect of AU on cell proliferation ability, different concentrations of AU ranging from 0 to 5 mM were prepared and tested.

### Quantitative real-time PCR

Logarithmically growing cells in a stable state were seeded in six-well plates at a density of 1 × 10^6^ cells per well. HFLS cells were divided into two groups: a control group without treatment and a group treated with 16 µM AU solution. PC12 cells were divided into three groups with different concentrations of AU treatment: 10 µM, 500 µM, and 5 mM, as well as a control group. All groups were incubated for 24 hours. Total RNA was extracted from the cells in each group using TRIzol Reagent (Cwbio, China) and reverse transcribed into cDNA using the SYBR Green Master Mix kit (TransGen Biotech, China). Human GAPDH was used as an endogenous control, and the primer sequences are listed in **Table 1**. Data were analyzed using the comparative Ct method (2-△△Ct).

**Table 1:** Primer sequence.

Primer	Sequence (5’-3’)
human-AURKA-F	GGGTGGTCAGTACATGCTCC
human-AURKA-R	GGCTCCCTCTGTTACAAAGTCA
RAT-AURKA-F	GCGAATGCTTTGTCCTACTGC
RAT-AURKA-R	CATCCGACCTTCAATCATCTCC
human-BTN3A2-F	GGCAGGTGGTGAACGTGTATG
human-BTN3A2-R	ACTTCGACGTGAAGATTAGAACCC
rat-BTN3A2-F	TAGGCACCAACGGCATTTC
rat-BTN3A2-R	CAACATAGGCCCAATACCCAC
human-CXCL10-F	TAGAACTGTACGCTGTACCTGC
human-CXCL10-R	TGTAGCAATGATCTCAACACG
rat-CXCL10-F	CTGCACCTGCATCGACTTCC
rat-CXCL10-R	CTTCTTTGGCTCACCGCTTTC
human-ERAP2-F	GCTGCTGAACTCTTCTCCC
human-ERAP2-R	TCCTGATGCTTGCTCGTT
human-MARCO-F	GGGACAATTTGCGATGACGA
human-MARCO-R	GGCCCTTCCTTTGGAGTAAC
rat-MARCO-F	GCACGTCCCAAAACACACAT
rat-MARCO-R	ACTTGCTGACGCAGTTGCTC
human-NeuN-F	GCCCGAGTGATGACCAACAAGAAG
human-NeuN-R	GTGGCGCAGCCCGAAATGTA
rat-NeuN-F	CCGTTTGCTTCCAGGGTCG
rat-NeuN-R	GCCGATGGTATGATGGTAGGGAT
human-MAP-2-F	GCCAGGCAGTGATTACTATGA
human-MAP-2-R	GATGGATAACTCTGTGCGAGA
rat-MAP-2-F	CTTGCCTATGTCTTGCCTTGA
rat-MAP-2-R	TCCATCGTTCCGCTAGTGTT
human-βIII -tubulin-F	GCCACGCTGTCCATCCACCA
human-βIII -tubulin-R	CGAAGCCGGGCATGAAGAAGT
rat-βIII -tubulin-F	CATCAGCAAAGTGCGTGAGGAG
rat-βIII -tubulin-R	GACAGGGTGGCGTTGTAGGG
human-GAPDH-F	AATCCCATCACCATCTTCCA
human-GAPDH-R	AAATGAGCCCCAGCCTTCT
rat-GAPDH-F	GGAAAGCTGTGGCGTGAT
rat-GAPDH-R	TCCACAACGGATACATTGGG

### Statistical analysis

R version 4.0.2 and GraphPad Prism 8.0 software were used for statistical analysis and visualization. One-way ANOVA was used to compare groups of samples in multiple groups, with the assumption of normality and homogeneity of variances. The significance level was set at α = 0.05, and a *P* value < 0.05 was considered statistically significant.

## Results

### Identification of DEGs

**Figure 2:**
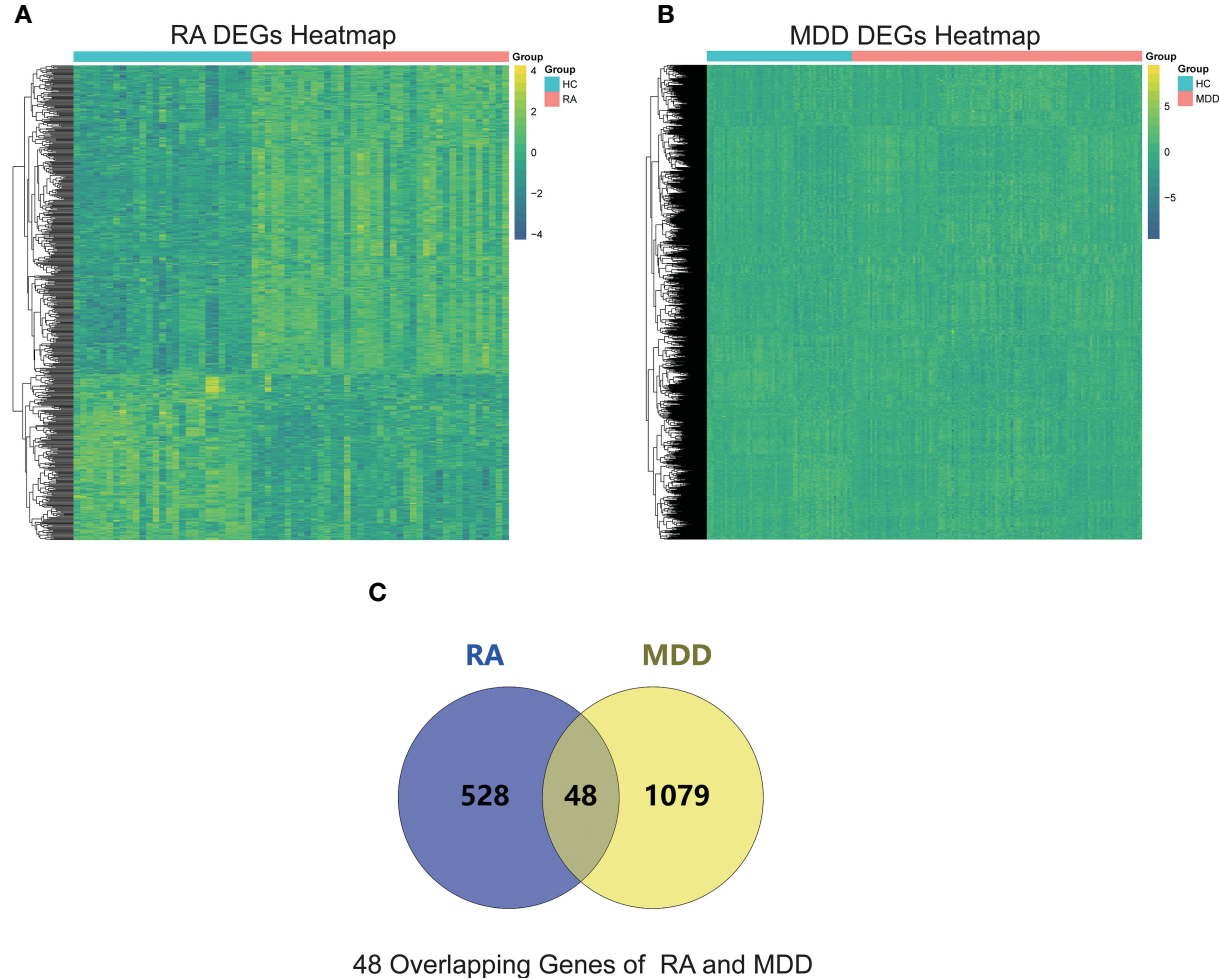
RA and MDD DEGs analysis. **(A)** A heat map of RA DEGs analysis results based on the merged GSE55235, GSE55457, and GSE77298 datasets. **(B)** A heat map of MDD DEGs analysis results based on the GSE98793 dataset. **(C)** Identification of 48 overlapping genes between the DEGs of RA and MDD.

The RA datasets from the GEO dataset contained 12403 genes in 64 synovium samples from 39 RA patients and 25 healthy individuals. A total of 576 genes were identified as RA-related DEGs in the datasets, of which 201 were downregulated and 375 were upregulated, as shown in a heatmap (**Figure 2A**). We obtained 1127 MDD-related DEGs in the GSE98793 dataset, of which 477 genes were downregulated and 650 genes were upregulated, as shown in a heatmap (**Figure 2B**). The 48 common genes between RA- and MDD-related DEGS are indicated by the Venn diagram (**Figure 2C**).

### Identification of co-expression gene modules

**Figure 3:**
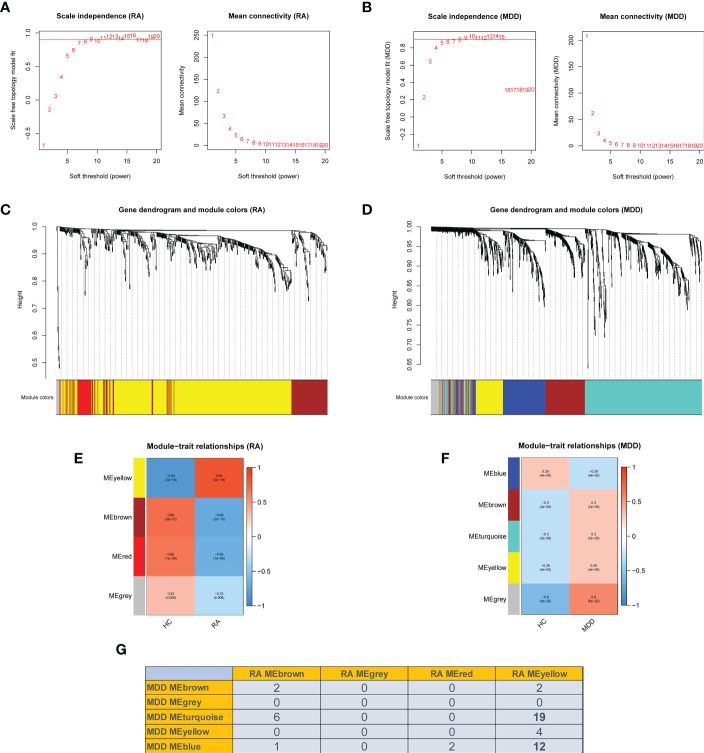
Weighted co-expression network related dataset construction and identification of related key modules in RA and MDD. **(A, B)** Analysis of network topology for various soft thresholds (β). The left panel shows the scale-free fit index (scale independence, y-axis) as a function of the soft threshold power (x-axis); the right panel displays the mean connectivity (degree, y-axis) as a function of the soft threshold power (x-axis). **(C, D)** Gene dendrograms were obtained by average linkage hierarchical clustering. The colored row underneath the dendrogram shows the module assignment determined by the dynamic tree cut method. **(E, F)** Module-trait relationships. Each row in the heatmap corresponds to an ME and each column to a clinical trait. Each cell contains the corresponding correlation and *p*-value. **(G)** Number of intersecting genes in related key modules in RA and MDD.

We performed WGCNA to identify co-expressed gene profiles in 39 RA datasets, 128 MDD datasets, and 91 healthy individual datasets. First, we divided the dataset samples from different sources into two groups with no detected outliers according to disease type: disease group (RA or MDD) and healthy control group (HC). Then, 8 and 5 were chosen as the optimal soft-threshold power β for the RA and MDD datasets, respectively, based on the scale independence of R2 greater than 0.9 and the mean connectivity tending to 0 to ensure a biologically meaningful scale-free network (**Figures 3A, B**).

Genes in the RA dataset were clustered into four modules, and the MDD dataset was clustered into five modules through hierarchical clustering analysis and dynamic branch cut methods for the gene dendrograms (**Figures 3C, D**). To identify the key modules related to RA and MDD, GS and MM were calculated to relate the modules to clinical traits. MM was defined as the correlation between gene expression values and module eigengene (ME). GS was defined as the correlation between genes and samples, as shown in **Figures 3E, F**. **Figures 3E–G** shows four RA modules and five MDD modules obtained using WGCNA. Two MDD-related modules (MDD-MEturquoise and MDD-MEblue) and RA-MEyellow shared 19 and 12 genes, respectively. Most DEGs (65%) found in RA and MDD were concentrated in these modules. Therefore, these modules can be considered as co-expressed gene modules closely related to RA with comorbid MDD.

### The PPI network key genes

**Figure 4:**
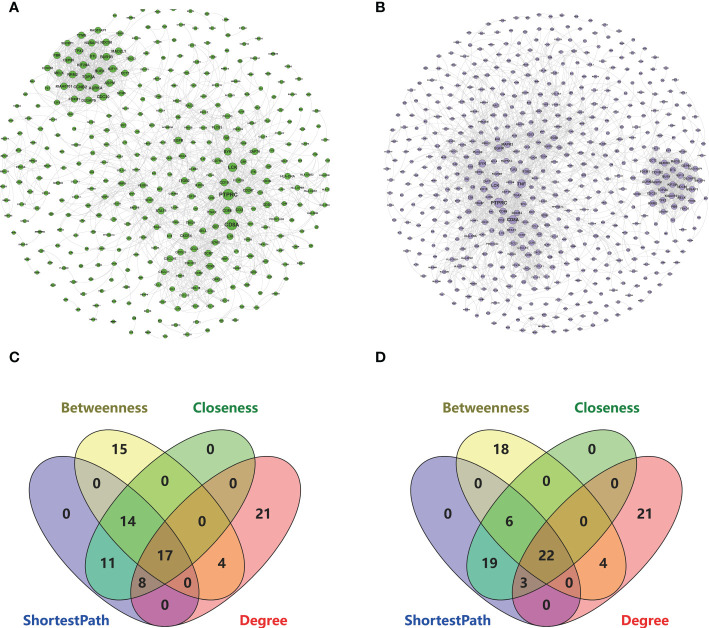
Based on the STRING database, 26 key genes were identified based on the two PPI network analysis in RA and MDD. **(A)** The left PPI network A is composed of genes in RA-MEyellow module and MDD-MEturquoise module (degree shown by node size). **(B)** The right PPI network B is composed of genes in RA-MEyellow module and MDD-MEblue module (degree shown by node size). **(C)** Screening of 17 key genes in the left PPI network A based on ShortestPath, Betweenness, Closeness, and Degree. **(D)** Screening of 22 key genes in the right PPI network B based on ShortestPath, Betweenness, Closeness, and Degree.

The interaction data of 943 genes that were composed of all genes in the co-expressed gene modules (RA-MEyellow and MDD-MEturquoise) were obtained from the STRING database and imported into Cytoscape to visualize the PPI network (**Figure 4A**). Similarly, a total of 644 genes in the co-expressed gene modules (RA-MEyellow and MDD-MEblue) were merged, and the interaction data were imported into Cytoscape to visualize the PPI network (**Figure 4B**). Based on the four network properties, we removed the relative nodes (network degree value < 5). All remaining nodes were screened to obtain the top 50 important nodes for each network property in the two PPI networks. Finally, we obtained 17 top genes of the co-expressed gene modules (RA-MEyellow and MDD-MEturquoise) and 22 top genes of the co-expressed gene modules (RA-MEyellow and MDD-MEblue) at the intersection of the Venn diagram. Twenty-six top genes as hub genes of RA with comorbid MDD based on PPI network analysis were obtained after the merger, as shown in **Figures 4C, D**.

### GO and KEGG pathway enrichment analysis

**Figure 5:**
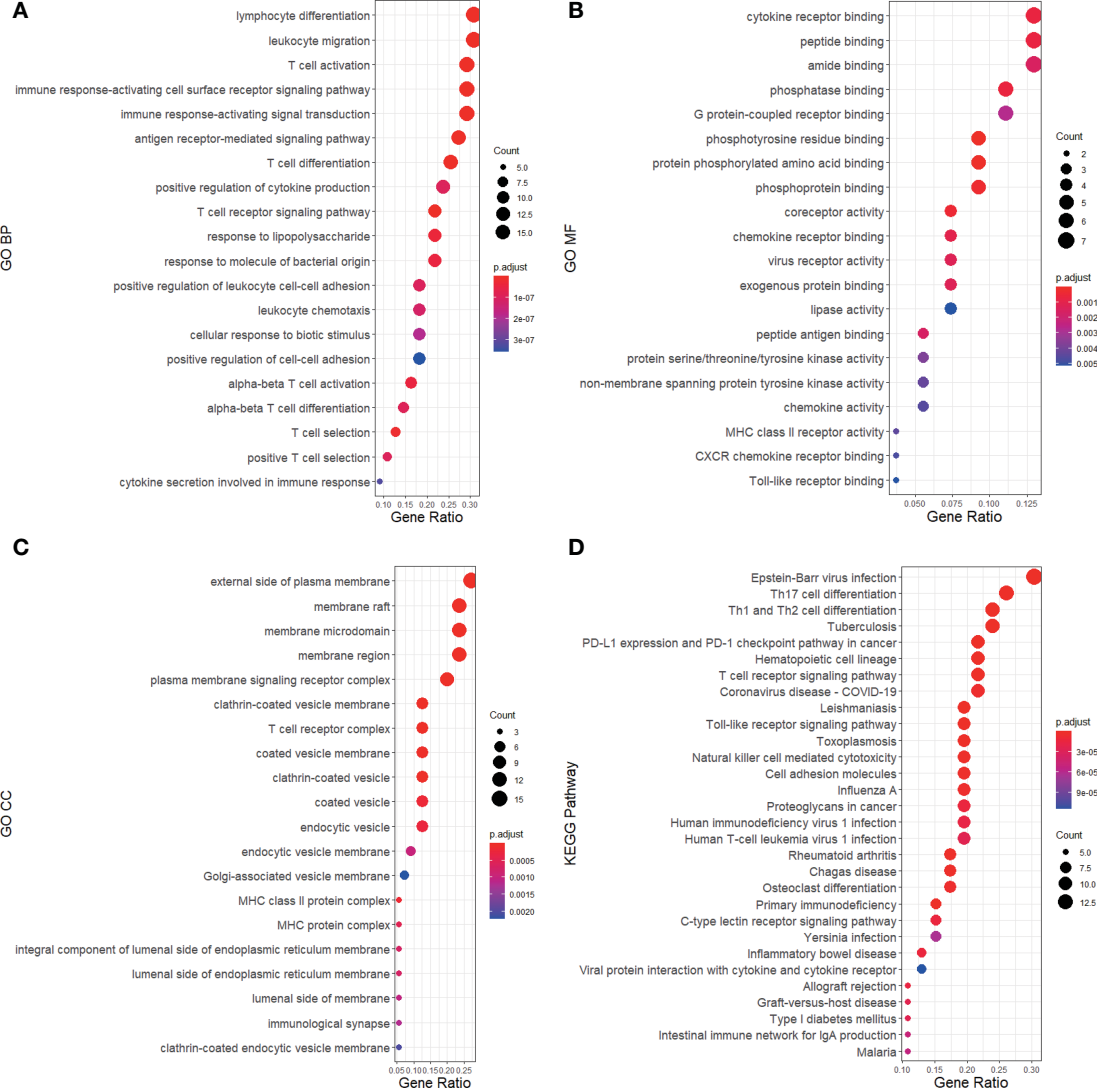
GO and KEGG pathway annotation for RA and MDD 55 core genes merged from 48 DEGs and 26 key genes of PPI network. The size of the ball represents the number of genes, and the color change of the ball corresponds to different *p*-values. **(A)** The first 20 significantly enriched GO annotations of BP. **(B)** The first 20 significantly enriched GO annotations of MF. **(C)** The first 20 significantly enriched GO annotations of CC. **(D)** The first 30 significantly enriched KEGG pathways.

Based on the 48 DEGs shared by RA and MDD, we added the 26 key genes obtained from the PPI network analysis and finally obtained 55 genes as the core genes for RA associated with MDD. GO enrichment was analyzed using the clusterProfiler package in R (**Figures 5A–C**). The results of these analyses showed that 705 GO entries were obtained in this study, including 611 biological process (BP), 54 molecular functions (MF), and 39 cellular components (CC). Regarding BP, the core genes were mainly enriched in lymphocyte differentiation (GO:0030098), leukocyte migration (GO:0050900), T cell activation (GO:0042110), immune response-activating cell surface receptor signaling pathway (GO:0002429), and immune response-activating signal transduction (GO:0002757). As for the MF, the core genes were mainly enriched in cytokine receptor binding (GO:0005126), peptide binding (GO:0042277), amide binding (GO:0033218), phosphatase binding (GO:0019902), G protein-coupled receptor binding (GO:0001664). Finally, regarding CC, the core genes were mainly enriched in the external side of the plasma membrane (GO:0009897), membrane raft (GO:0045121), membrane microdomain (GO:0098857), membrane region (GO:0098589), and plasma membrane signaling receptor complex (GO:0098802).

A KEGG pathway analysis was performed (**Figure 5D**). The core genes were mainly focused on 89 pathways. The pathways in the KEGG enrichment analysis were related to Epstein-Barr virus (EBV) infection (hsa05169), Th17 cell differentiation (hsa04659), Th1 and Th2 cell differentiation (hsa04658), tuberculosis (hsa05152), PD-L1 expression, and the PD-1 checkpoint pathway in cancer (hsa05235). We found that RA and MDD share many molecular mechanisms.

### Receiver operating characteristic curve analysis of diagnostic markers

**Figure 6:**
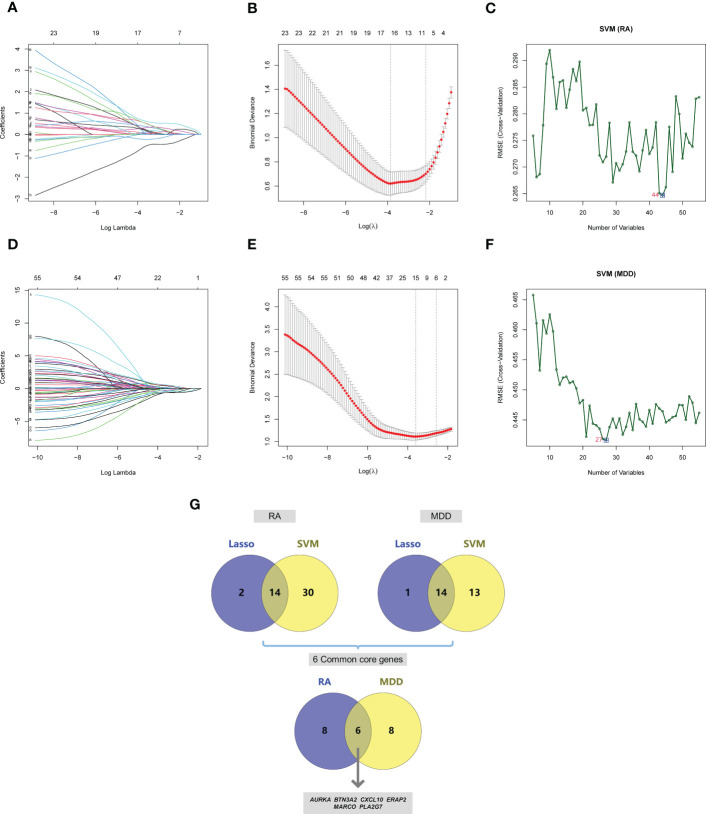
Screening of core genes and validation based on machine learning methods in RA and MDD. **(A)** Coefficient profiles of variables in the LASSO regression model in RA. **(B)** Ten-fold cross-validation for turning parameter (*λ*) selection in the LASSO regression model in RA. **(C)** The optimum root mean squared error (RMSE) of SVM-based method based on 44 characteristic genes in RA. **(D)** Coefficient profiles of variables in the LASSO regression model in MDD. **(E)** Ten-fold cross-validation for turning parameter (*λ*) selection in the LASSO regression model in MDD. **(F)** The optimum root mean squared error (RMSE) of SVM-based method based on 27 characteristic genes in MDD. **(G)** 14 core genes in RA and 14 core genes in MDD screened by LASSO regression model and SVM-based method, and 6 common core genes were obtained after taking the intersection.

Based on the 55 core genes of RA associated with MDD, the LASSO regression model and SVM-based method were used to screen diagnostic markers related to disease diagnosis. As shown in **Figures 6A–C**, following the 10-fold cross-validation procedure, LASSO regression identified 16 diagnostic core genes of RA in the model. The other 44 diagnostic core genes of RA were screened by SVM-based method. As shown in **Figures 6D–F**, 15 and 27 MDD diagnostic core genes from the core genes were also identified by LASSO regression and SVM, respectively. The common diagnostic core genes of these two diseases are considered diagnostic markers for RA with MDD. As shown in **Figure 6G**, six diagnostic markers were obtained: *AURKA*, *BTN3A2*, *CXCL10*, *ERAP2*, *MARCO* and *PLA2G7*.

**Figure 7:**
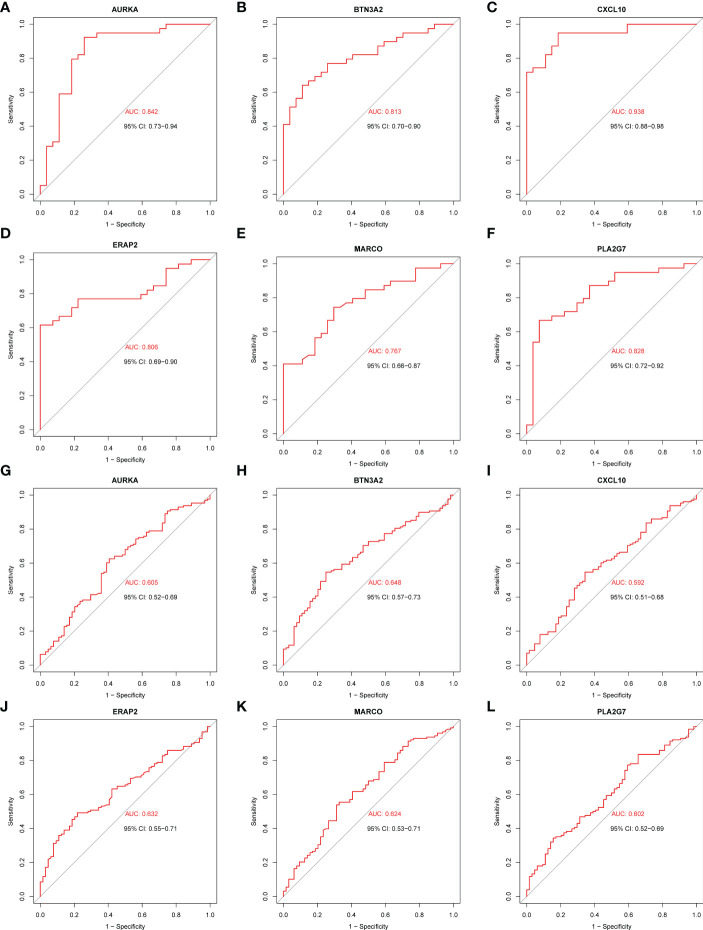
ROC curves of the 6 common core genes in RA and MDD. **(A, B, C, D, E, F)** ROC curves of *AURKA*, *BTN3A2*, *CXCL10*, *ERAP2*, *MARCO*, *PLA2G7* in the RA dataset, respectively. **(G, H, I, J, K, L)** ROC curves of *AURKA*, *BTN3A2*, *CXCL10*, *ERAP2*, *MARCO*, *PLA2G7* in the MDD dataset, respectively.

We drew the receiver operating characteristic (ROC) curve of the diagnostic markers in RStudio to determine their diagnostic value. The results showed that most of diagnostic markers (**Figure 7**) had significant diagnostic value in the disease classification. However, their prediction performance in the RA dataset was much better than in the MDD dataset, which may be attributed to the fact that MDD is a mental disease that rarely leads to organ lesions or inflammation.

### Immune cell correlation analysis

**Figure 8:**
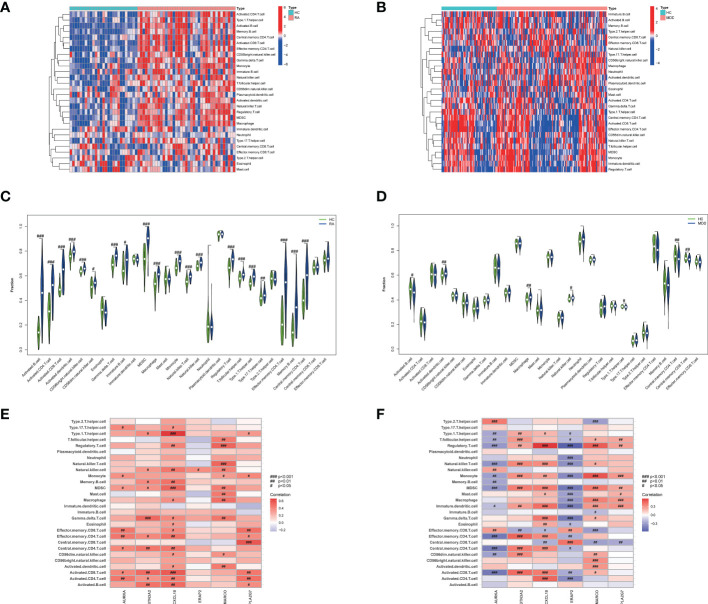
The 28 immune cells and their correlation with the 6 common core genes in RA and MDD. **(A)** Heatmap of 28 immune cell expression scores in RA. **(B)** Heatmap of 28 immune cell expression scores in MDD. **(C)** Comparison of 28 immune cells in samples with HC and RA by Fraction. **(D)** Comparison of 28 immune cells in samples with HC and MDD by Fraction. **(E)** Spearman correlation analysis of the 6 common core genes and 28 immune cells in RA. **(F)** Spearman correlation analysis of the 6 common core genes and 28 immune cells in MDD. #*p* < 0.05; ##*p* < 0.01; ###*p* < 0.001.

The results of ssGSEA showed that in RA, the scores of immune cell content were higher in most RA groups but lower in the control group, and 20 out of 28 immune cells (activated B cells, activated CD4 T cells, activated CD8 T cells, activated dendritic cells, CD56 bright natural killer cells, CD56 dim natural killer cells, Gamma delta T cells, immature B cells, MDSCs, macrophages, monocytes, natural killer T cells, natural killer cells, regulatory T cells, T follicular helper cells, type 1 T helper cells, type 17 T helper cells, effector memory CD4 T cells, memory B cells, and central memory CD4 T cells) were significantly different between the two groups, as shown in **Figures 8A, C**. However, the immune cell content scores were lower in most MDD groups, except for activated B cells, activated dendritic cells, macrophages, natural killer cells, type 1 T helper cells, central memory CD4 T cells, and central memory CD8 T cells. There was no significant difference in the number of other immune cells between the two groups, as shown in **Figures 8B, D**.

We found that the levels of six immune cell types (activated B cells, activated dendritic cells, macrophages, natural killer cells, type 1 T helper cells, and central memory CD4 T cells) were significantly different in both RA and MDD, as shown in **Figures 8E, F**. In RA, *CXCL10* and *MARCO* are closely related to the content of various immune cells, while in MDD, except for *PLA2G7*, the expression levels of the other five diagnostic markers are correlated with the content of most immune cells.

### In silico validation of the targets using molecular docking

According to the ADMET evaluation, AU exhibits many of the qualities of an ideal reagent for drug-like qualities (Lipinski), water solubility (solubility), lipophilicity (LogP), and other parameters. However, as shown in **Table 2**, the intestinal absorption (GI absorption) and oral availability (bioavailability) were low. Thus far, there has been no noted toxicity (hERG, AMES Toxicity, Skin Sensitization).In this study, we selected three target proteins (*AURKA*, *ERAP2*, and *PLA2G7*) for molecular docking analysis to predict their potential therapeutic effect on patients with RA and MDD.

**Table 2:** ADMET properties of AU by ACD/Labs, SwissADME and ADMETlab 2.0.

Name	*Aucubin* (AU)	Source
**PubChem CID**	91458	PubChem
**Molecular Formula**	C15H22O9	
**CAS**	479-98-1	
**MW**	346.33	SwissADME
**TPSA**	149.07	
**Lipinski (violations)**	1 (NHorOH > 5)	
**GI absorption**	Low	
**Log P**	Hydrophilic	ACDLabs
**Solubility**	Soluble	
**BBB permeant**	No	
**Pgp substrate**	Yes	
**Bioavailability (%) (Dose, mg = 50.00)**	3.17	
**hERG**	Non-inhibitor	
**AMES Toxicity (Probability value)**	0.1-0.3	ADMETlab 2.0
**Skin Sensitization (Probability value)**	0.0-0.1	

**Figure 9:**
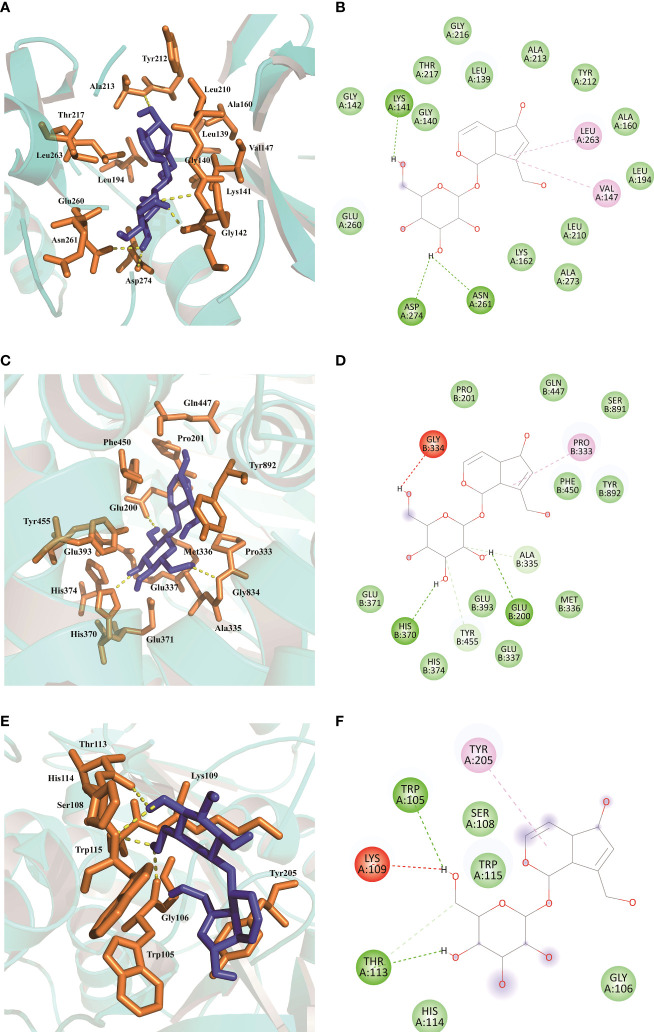
Molecular docking results of AU interaction with *AURKA*, *ERAP2* and *PLA2G7*. **(A, B)** Molecular docking conformation of AU interaction with *AURKA*. **(C, D)** Molecular docking conformation of AU interaction with *ERAP2*. **(E, F)** Molecular docking conformation of AU interaction with *PLA2G7*.

The total kollman charges for *AURKA*, *ERAP2*, and *PLA2G7* were added as -130.535, -451.539, -190.286. The hydrogen atoms and gasteiger charge for AU (0.0002) were added and saved in the pdbqt format. In this paper, molecular docking took the binding sites of the original ligands as the reference binding sites, and the specific information was shown in **Table 3**. The docking scores between AU and *AURKA* were -7.7 (kcal/mol). As shown in **Figures 9A, B**, AU interacted with *AURKA* by forming a hydrogen bond with Lys141, Asp274, Asn261, and interacted with the surrounding residues by forming other bonds. The docking scores of AU and *ERAP2* were -8.4 (kcal/mol). As shown in **Figures 9C, D**, AU interacted with *ERAP2* by forming a hydrogen bond with Gly334, His370, Glu200, and interacted with surrounding residues by forming other bonds. The docking score between AU and *PLA2G7* was -6.0 (kcal/mol). As shown in **Figures 9E, F**, AU interacted with *PLA2G7* by forming a hydrogen bond with Trp105, Lys109, Thr113, and interacted with surrounding residues by forming other bonds.

**Table 3:** Summary of molecular docking details.

Targets	Grid dimensions (Å)	Center grid box			Number of poses generated	Affinity (kcal/mol)	
		center x	center y	center z		Aucubin	Original ligand
AURKA	30×30×30	-7.525	26,575	79.368	9	-7.7	-6.8
ERAP2	30×30×30	88.371	9.906	123.592	9	-8.4	-8.2
PLA2G7	30×30×30	25.469	4.174	-2.949	9	-6.0	-7.6

### *In vitro* validation of the targets using MTT assay and qPCR

**Figure 10:**
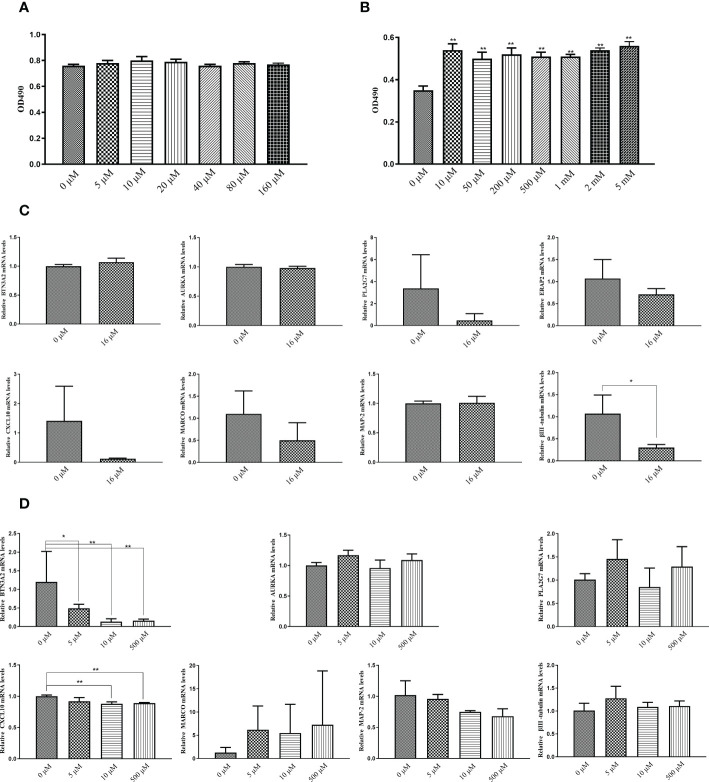
*In vitro* validation of the targets using MTT assay and qPCR. **(A)** MTT assay to measure cell viability in PC12 cells after treatment with AU at 0 to 160 μM concentrations (In comparison with the control group, *P < 0.05, and ** P < 0.01). **(B)** MTT assay to measure PC12 cell proliferation after treatment with AU at 0 to 5 mM concentrations (In comparison with the control group, *P < 0.05, and ** P < 0.01). **(C)** Quantitative analysis of *BTN3A2, AURKA, PLA2G7, ERAP2, CXCL10, MARCO, MAP-2*, and βIII-tubulin gene expression in HFLS cells by real-time PCR (In comparison between two groups, *P < 0.05, and ** P < 0.01). **(D)** Quantitative analysis of *BTN3A2, AURKA, PLA2G7, CXCL10, MARCO, MAP-2*, and βIII-tubulin gene expression in PC12 cells by real-time PCR (In comparison between two groups, *P < 0.05, and ** P < 0.01).

The results of the MTT assay indicated that the concentration of the test drug had no toxic effect on the cells within the range of 0-160 μM (**Figure 10A**). Upon adjusting the concentration range from 0-5 mM and reducing the cell density to 1 × 10^4^ cells/well, the MTT assay showed that AU significantly increased the proliferation of PC12 cells (**Figure 10B**). To further investigate the effect of AU at various concentrations on the gene expression levels of six diagnostic markers and MAP-2, βIII-tubulin, we examined the expression levels of these genes in PC12 and HFLS cells after 24 hours of AU treatment. In HFLS cells, the expression levels of the six diagnostic markers did not change significantly, but the expression level of βIII-tubulin was significantly downregulated (**Figure 10C**). In PC12 cells, the expression levels of *CXCL10* and *BTN3A2* were significantly downregulated following AU treatment (**Figure 10D**).

## Discussion

Patients with depression have a 14–48% chance of developing RA (43). Depression is the most common comorbidity associated with RA. However, it is frequently neglected and under-treated in clinical practice. Depression has various effects on the progression of RA, including disease activity, other arthritis-related comorbidities, pain levels, quality of life, and mortality, all of which lead to worse clinical outcomes. Furthermore, RA and depression initiate a vicious cycle that exacerbates the other symptoms. This strong association between depression and RA is already partly explained by the assumption of a model based on the hypothesis of inflammation and crosstalk between the central, peripheral, and immune systems. The management of individuals with dual diagnoses should be closely monitored to avoid undue distress.

Gene expression variations and patterns shed light on the mechanism of RA comorbidity with depression and may aid in the identification of targets for therapeutic intervention. In this study, we used WGCNA to construct network hierarchical clustering trees and co-expression modules associated with RA and depression. We obtained 26 key genes of RA that were associated with MDD based on PPI network analysis. In functional enrichment analysis, some of these shared molecular mechanisms have been experimentally validated, and some of them, such as tuberculosis, are reported for the first time.

It has been reported that Epstein-Barr virus (EBV) infection promotes autoimmunity, and in many studies, the evidence for whether EBV infection is causal of autoimmunity appears high (44). A study published in 1970 showed that there were quantitative differences in EBV protein antibodies in RA patients, and that the route of EBV infection may be closely related to the occurrence and development of RA and MDD complications (45). EBV is a double-stranded DNA virus belonging to the herpes family. The globally prevalent EVB virus has significant effects on the immune system and is considered an attractive candidate pathogen for RA. EVB can be latent in the B lymphocyte and the salivary gland epithelium for a long time, with a lifetime prevalence of 90%. Evidence of *in vitro* EBV infection was observed in the lymphocytes of RA patients and antibodies to EBV antigens were significantly increased in their serum. As a polyclonal activator of B cells, EVB virus can induce rheumatoid factor and autoantibody production *in vitro* and *in vivo*. These immunopathological events may explain the link between EVB virus and RA disease (46). Children with EBV infection are at high risk of depression in adulthood (47).

Lymphopenic mice demonstrate that an adaptive immune system composed of T cells and B cells can be a potential factor in depression. T helper (Th) cells differentiate into different lineages under the influence of cytokine environment, antigen stimulation, and co-stimulation. A decrease in regulatory T cells and an increase in Th17 cells were observed in patients with depression. The discovery that Th17 cells are involved in depression evolves from the classic theory that Th17 cells produce inflammatory cytokines IL-17A and IL-6, which is required for differentiation, and contribute to depression onset and maintenance (48). Increased physiological levels of IL-7 affect joint inflammation, osteoclastogenesis, and neovascularization associated with autoimmune diseases. The increased content of IL-7 in the synovial tissue and fluid of RA allows monocytes to enter the inflamed joints to form macrophages and mature osteoclasts (49).

The chronic immune response in RA may be driven by activated Th1 cells without sufficient Th2 cell differentiation to downregulate inflammation. The combined effect of Th1 cell activation-driven cascades and the inability of Th2 cell differentiation to downregulate the inflammation is the underlying mechanism of chronic immune responses in RA. Th1 cells infiltrating the synovium can secrete abundant proinflammatory cytokines and induce macrophage and neutrophil infiltration (50). PD-1 is an immunosuppressive molecule that inhibits inflammatory responses. It controls the inflammatory activity of T cells by adjusting the immune system’s response to human cells, helping to improve immunotolerance. Therefore, impairment of the PD-1/PD-L1 pathway is considered to play an important role in many immune-mediated diseases including RA (51).

To further explore the diagnostic markers of RA complicated by MDD, six diagnostic markers were obtained from 55 core genes based on the two algorithms. *AURKA* encodes a cell cycle-regulated kinase that appears to play a role in microtubule formation and/or spindle pole stabilization during chromosome segregation. *BTN3A2* is the gene most closely connected with treatment response according to a genome-wide methylation analysis of DNA in RA patients receiving anti-rheumatic therapy for the first time (52). *BTN3A2* has also shown a pleiotropic association with MDD (53). TNF stimulates neurons to produce *CCL2*, *CCL7*, and *CXCL10*. These chemokines are closely related to RA and depression by interfering with the microglial elongation process (54). MDX-1100, an anti-*CXCL10* monoclonal antibody, had demonstrated well tolerated and clinically effective in patients with RA who had an inadequate response to methotrexate (55). This further confirms that *CXCL10* plays a role in the immunopathogenesis of RA. The *ERAP2* gene has been shown to be expressed considerably more in the CD4 + T cells of patients with RA who react to glucocorticoid medication, suggesting that *ERAP2* may be a clinical predictor of response to glucocorticoid therapy in patients with RA (56). *MARCO*, a macrophage receptor with a collagen structure, is involved in the uptake of apoptotic cells, and the ability of macrophages to promptly clear apoptotic cells has been linked to autoimmune diseases (57). Lower levels of the platelet-activating factor acetyl hydrolase, a protein encoded by *PLA2G7*, may result in a loss of anti-inflammatory function, triggering juvenile RA (58). The relationship between the immune cell types and the diagnostic markers of RA were evaluated using ssGSEA which showed that the six candidate diagnostic genes of RA complicated by MDD were correlated with immune cell content to varying degrees.

The long onset time of RA is a serious threat to human health and quality of life. In recent years, there have been many applications of natural product in arthritis. AU with antioxidant, anti-inflammatory, neuroprotective, and osteoprotective properties are high-profile natural small molecules. AU has a wide range of biological effects and is a compound with rich potential sources, a good safety profile, and many beneficial biological activities. It has high application potential in health products and medicines, and can be used to treat RA, depression, hypertension, lower back pain, and other diseases (59). In animal models of neurological diseases, AU inhibits the activation of glial cells, which are responsible for brain inflammation (60, 61). Modern medical research has demonstrated that AU can increase the biomechanical quality of the femur, bone mineral density, and bone microarchitecture to prevent osteoporosis (62). In a molecular docking study, three target proteins (*AURKA*, *ERAP2*, and *PLA2G7*) predicted the potential therapeutic effect of AU on RA with MDD.

*BTN3A2* expression has been found to be increased in patients with RA, and inhibiting *BTN3A2* may improve RA symptoms in animal models (63). Elevated levels of *CXCL10* have been associated with impaired cognitive performance in patients with depression (64), and may accelerate disease progression in RA patients (65). *In vitro* studies suggest that AU may exert therapeutic effects by decreasing the expression of *CXCL10* and *BTN3A2*. There is a growing body of evidence supporting the role of adult neurogenesis in the pathology and physiology of brain homeostasis and depression (66). AU’s effect on PC12 cell proliferation may be one mechanism by which it improves depression. HFLS cells, found in the synovial lining of joints, may become activated and produce abnormal amounts of pro-inflammatory cytokines and matrix metalloproteinases (MMPs) in RA patients, potentially leading to joint damage and inflammation (67). βIII-tubulin has been shown to positively regulate the activation of HFLS cells (68), and decreased βIII-tubulin gene expression suggests that AU may inhibit activation of HFLS cells in RA patients.

In conclusion, 55 core genes are likely to be involved in the mechanism underlying RA with MDD, which predicts multiple therapeutic pathways closely related to the disease. Six diagnostic markers not only affect immune cells but are also potential therapeutic targets for RA with comorbid MDD.

## Data availability statement

The datasets presented in this study can be found in online repositories. The names of the repository/repositories and accession number(s) can be found in the article/supplementary material.

## Author contributions

J-JS was responsible for data collection and sorting, computational modeling, and analysis; T-TZ wrote the research paper; J-YW provided research guidance for writing the research paper; L-DT participated in data analysis; YY provided research guidance on rheumatoid arthritis; LZ provided the design and general guidance of the research framework. J-JS and T-TZ contributed equally to this work and should be considered as co-first authors. J-YW and LZ contributed equally to this work and should be considered as co-corresponding authors. All authors contributed to the article and approved the submitted version.
